# Vinasse fertirrigation alters soil resistome dynamics: an analysis based on metagenomic profiles

**DOI:** 10.1186/s13040-017-0138-4

**Published:** 2017-05-23

**Authors:** Lucas P. P. Braga, Rafael F. Alves, Marina T. F. Dellias, Acacio A. Navarrete, Thiago O. Basso, Siu M. Tsai

**Affiliations:** 1 Cell and Molecular Biology Laboratory, Center for Nuclear Energy in Agriculture (CENA), University of São Paulo (USP), Av. Centenário 303, Piracicaba, 13400-970 São Paulo Brazil; 2 Brazilian Bioethanol Science and Technology Laboratory (CTBE), University of Campinas (Unicamp), Campinas, Brazil; 3 Department of Chemical Engineering, Polytechnic School, University of São Paulo, São Paulo, Brazil

**Keywords:** Sugarcane, Vinasse, Resistome, Antibiotics, Resistance genes, Metagenomic profiles

## Abstract

Every year around 300 Gl of vinasse, a by-product of ethanol distillation in sugarcane mills, are flushed into more than 9 Mha of sugarcane cropland in Brazil. This practice links fermentation waste management to fertilization for plant biomass production, and it is known as fertirrigation. Here we evaluate public datasets of soil metagenomes mining for changes in antibiotic resistance genes (ARGs) of soils from sugarcane mesocosms repeatedly amended with vinasse. The metagenomes were annotated using the ResFam database. We found that the abundance of open read frames (ORFs) annotated as ARGs changed significantly across 43 different families (*p*-value < 0.05). Co-occurrence network analysis revealed distinct patterns of interactions among ARGs, suggesting that nutrient amendment to soil microbial communities can impact on the coevolutionary dynamics of indigenous ARGs within soil resistome.

**Electronic supplementary material:**

The online version of this article (doi:10.1186/s13040-017-0138-4) contains supplementary material, which is available to authorized users.

Vinasse is a by-product of the sugar-ethanol industry produced in large quantities [1], and it is chemically composed of water, organic matter, and mineral elements [2]. It derives from the distillation process of the fermented broth, and it is obtained after the fermentation step in fuel ethanol production from sugarcane [3]. Since the 1960’s, application of vinasse *in natura* has been used as fertilizer in the sugarcane fields of Brazil to resolve the ecological problem of its disposal within the environment, a process known as “fertirrigation” [2].

However, recent research has demonstrated that the utilization of organic compounds as fertilizers in agriculture soils can alter the selective pressure that drives antibiotic resistance genes (ARGs) on soil-borne microbial communities [4]. Soil ecosystems are considered to harbor a remarkable diversity of ARGs [4]. According to Wright et al. [5], the collection of all the ARGs and their precursors in both pathogenic and non-pathogenic bacteria can be defined by the term ‘resistome’. The number of studies reporting the dynamics of resistome in the environment is growing [4]. To date, the impacting activities often reported are associated to manure fertilization and wastewater utilization in agricultural practices [6, 7]. The discovery of new sources of anthropogenic practices that may affect ARGs in the environment is a challenging and timely topic, since the mechanisms regulating the spread of resistance and evolution of pathogens in environmental resistome are widely unknown [8].

Within this context, we hypothesized that vinasse fertirrigation can impact the soil resistome dynamics. Therefore, the main objective of the present study was to evaluate the impact of vinasse amendment on the abundance of ARGs from soil resistome by analyzing metagenomic datasets from a previous study [9]. In the referred study, a greenhouse experiment was performed in which vinasse was repeatedly applied to sugarcane-cultivated soils in plastic pots (100 L) filled with 90 kg of soil (*n* = 3). Vinasse applications were supplemented with urea (450 g N kg-1) at a rate of 60 kg N ha-1 as normally performed in sugarcane field production [9]. Therefore we compared the pots which received vinasse (V+) against pots which received only urea (V-). Before starting the experiment, all the pots were treated with mineral fertilizers (150 kg ha-1 of P_2_O_5_ and 80 kg ha-1 of KCl) following the basic recommendations for sugarcane field production. Bulk soil samples were collected at the 7^th^ day after vinasse application (7^th^, 157^th^ and 217^th^ days after planting, dap). Vinasse samples used in the experiment were obtained downstream from fermentation process in a sugar-ethanol mill (São Paulo - Brazil). Vinasse was applied to the soil at a rate of 0.06 L kg^-1^ (120 m^3^ ha^-1^) as a source of potassium (K) according to technical recommendations [10]. Therefore, to address the objective proposed in the present study, we used eighteen metagenomic datasets, considering the V+ and V- treatments sampled in three different periods (7^th^, 157^th^ and 217^th^ dap) (*n* = 3). The datasets were obtained using a MiSeq Personal Sequencing System (Illumina, San Diego, CA, USA) and are available through the Metagenomics Rapid Annotation (MG-RAST) server (http://metagenomics.anl.gov/linkin.cgi?project=10854). More details on how the metagenomic datasets were generated can be found in Navarrete et al. [9]. A total of 7,984,790 open reading frames (ORFs) assigned with PRODIGAL [11] were screened for ARGs using hmmscan from HMMER3 [12] and the hidden markov models (HMM) profiles available at the ResFam, a curated database of protein families confirmed for antibiotic resistance function [13]. The screening was performed using the default parameters for both tools and the count tables of ARGs obtained from the metagenomic datasets were analyzed using the software STAMP (Statistical Analysis of Metagenomic Profiles) [14]. The variance of significant biological importance was detected by using the Welch’s test (*p*-value < 0.05) and Welch’s inverted for calculating the confidence intervals of the effect sizes, which indicates the difference in proportions of sequences assigned to a given ARG. Error bar plots were generated to show the *p*-values and the effect sizes of ARGs found to be changed significantly (Fig. 1).

**Fig. 1:**
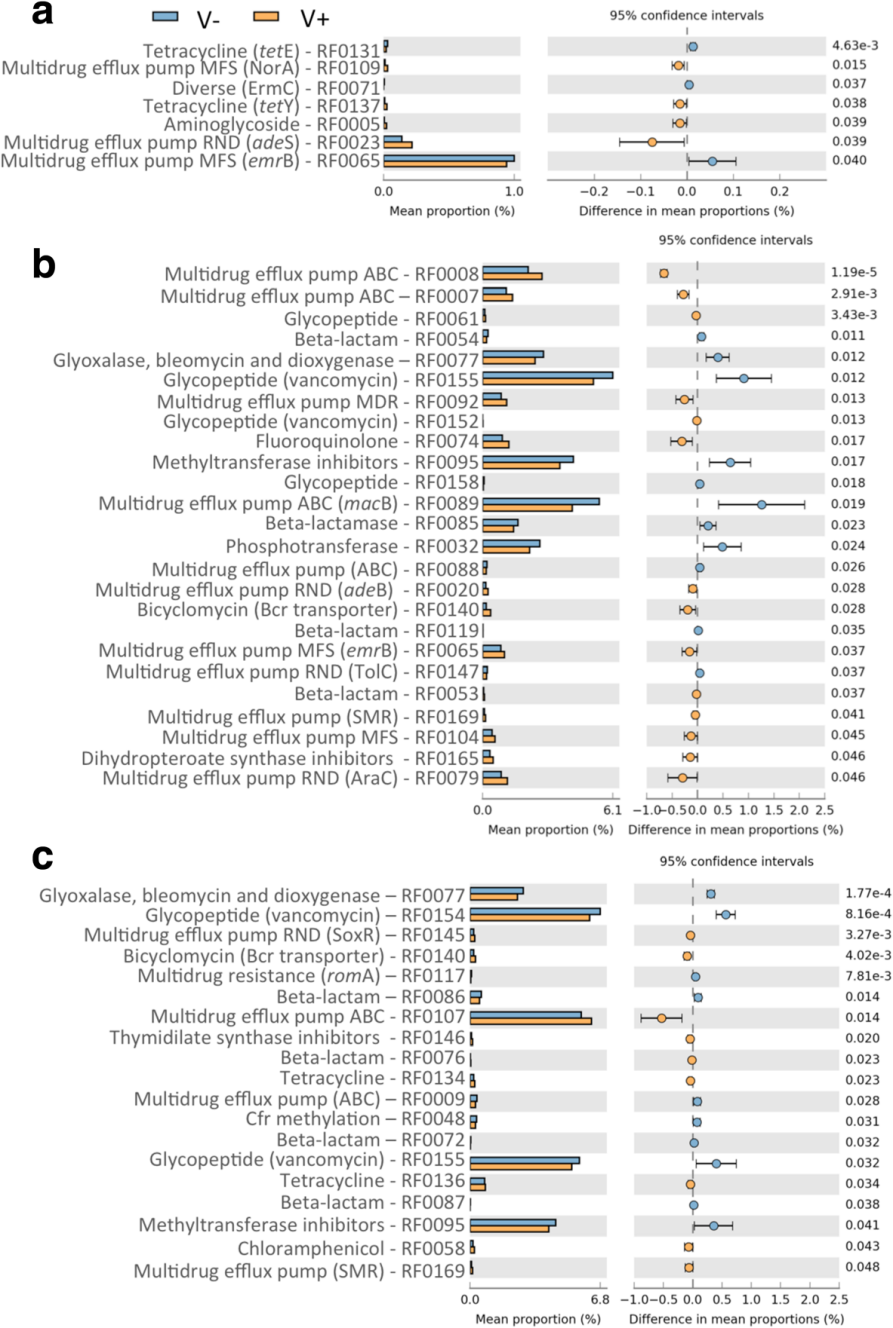
Soil resistome dynamics revealed by the response of antibiotic resistance genes (ARGs) to vinasse amendment. The error bar plots indicate the ARGs that changed abundance significantly (Welch’s test, *p*-value < 0.05) after vinasse amendment (**a**) 7 days after planting (dap), (**b**) 157 dap and (**c**) 217 dap. The ARGs are represented by the antimicrobial compound that they may antagonize and its respective ID from the RESFAM database. Orange bars represent the response in vinasse-amended treatment (V+) and blue bars represent the response in treatment without vinasse amendment (V-)

Next, in order to detect patterns of interaction among ARGs, we performed a network analysis. The models were built using the Co-occurrence Network inference tools (CoNet) [15] to detect strong and significant relationships among the different ARGs that were identified. Spearman (-0.8 > ρ > 0.8) and Pearson (-0.8 > r > 0.8) correlations with Steinhaus similarity index (>0.8) were the measures used to detect strong interactions. Relationships that were not detected by the three methods were not included in the network models. The *p*-values were computed by bootstrap (1000 interactions) based on a set of edge-specify score distribution [15], merged using the Brown method [16] and adjusted with the Benjamini-Hochberg procedure. Only relationships with *p*-values < 0.05 were kept in the models. The models were constructed separately for the compositions of ARGs found in V+ and V- across the three sampling points (7 dap, 157 dap and 217 dap), resulting in a total of 6 models. To evaluate the differences in the model structure we compared them by the change in the composition of nodes according to betweenness centrality (BC) (a parameter interpreted as the amount of influence that a node exert over the model [17]) and by the type of interactions whether negative or positive.

Our analysis reveals that vinasse amendment caused changes in the abundance of diverse ARGs. Although no antibiotic-specific pattern of resistance could be identified as a response to vinasse amendment, several genes associated to distinct families of multidrug efflux pumps were found to be enriched in V+ samples from 7, 157 and 217 dap (Fig. 1). Besides that, worth noting that genes associated with resistance against thymidylate synthase inhibitors (RF0146), dihydropteroate synthase inhibitors (RF0165) and bicyclomycin (RF0140) were found to be enriched in V+ at the 157^th^ dap or the 217^th^ dap. The later (RF0140) was found to be enriched at 157^th^ and 217^th^ dap (Fig. 1b-c). Further, vinasse amendment was found to change the patterns of co-occurrence within the community of ARGs as revealed by the results predicted in the network models (Fig. 2; Additional file 1: Table S1, Additional file 2: Table S2, Additional file 3: Table S3, Additional file 4: Table S4, Additional file 5: Table: S5 and Additional file 6: Table S6). The composition of nodes, ranked by the amount of control that they exert over the network (i.e., BC), was different in all the models (Additional file 7: Table S7).

**Fig. 2:**
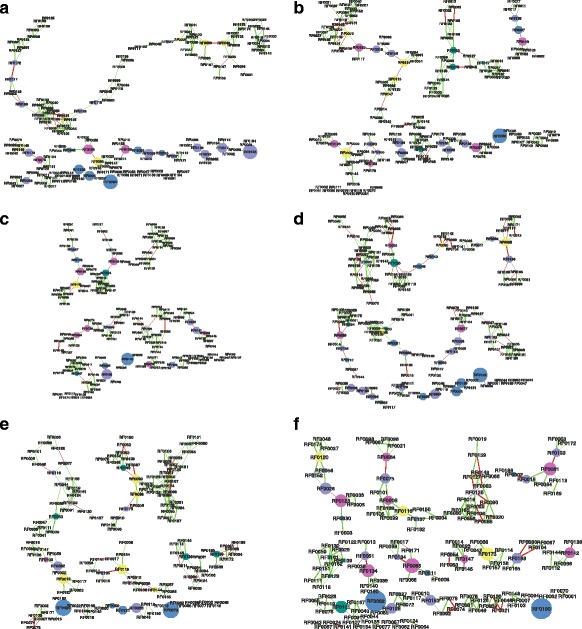
Ecological interactions within the communities of antibiotic resistance genes (ARGs) predicted by network models. The models were constructed using the ARGs profiles from conditions without vinasse (V-) and with vinasse (V+) detected at the 7^th^ day after planting (dap) **a**) (V-) and **b**) (V+), at the 157^th^ dap **c**) (V-) and **d**) (V+) and at the 217^th^ dap **e**) (V-) and **f**) (V+). The nodes size and color distinguishes their values of betweeness centrality (BC), the higher the BC the bigger the node, and their degree (number of connections), respectively

ARGs play important role in microbial ecology and evolution and are strongly associated with fitness, being responsible for detoxification by the exposure to antimicrobial compounds [4]. The results presented here indicate that vinasse applications to the soil gradually changed mechanisms of resistance (Fig. 1). Although diverse antibiotic-specific resistance genes were changed by vinasse amendments, the enrichment of multidrug efflux systems of different types such as resistance nodulation-nodulation-division (RND), major facilitator superfamily (MFS), small multidrug resistance (SMR) superfamily and ATP-binding cassette (ABC) transporters, suggests a possible large-scale effect of detoxification.

Some antimicrobial compounds, such as sulfonamides, can act targeting the enzyme dihydropteroate synthase which is involved in the folate cycle, an important pathway in cell metabolism [18]. Resistance mechanism against dihydropteroate synthase inhibitors (RF0165) was enriched in V+ (Fig. 1b). Likewise, dihydropteroate synthase, thymidilate synthase also participates on folate cycle and some drugs like 5-fluorouracil can target this enzyme. Mechanism of resistance against thymidylate synthase inhibitors (RF0146) was found to be enriched in V+ (Fig. 1c). The bicyclomycin resistance (RF0140), also enriched in V+ (Fig. 1b-c), is part of a family of multidrug antiporters, and was demonstrated to be involved in the protection of folate cycle [19]. Folate synthesis is essential to most of microbial cells and the interference on this pathway can be harmful leading to the inability of DNA replication [20]. Therefore, vinasse application to the soil might have disturbed the microbial capacity to synthetize essential metabolic compounds, and this would explain why mechanisms of protection to enzymes were found to be enriched in V+. This, together with the enrichment in several different types of multidrug efflux pumps, suggest a toxic effect of vinasse to soil microbes.

So far, the toxicity of vinasse to soil microbial community has not been evaluated. Vinasse applications follow agronomical recommendations taking into account plant nutritional requirements, soil characteristics and the mineral composition in vinasse. However, our results indicate that the effect of toxicity should be tested in further research. In addition, network analysis (Fig. 2; Additional file 7: Table S7) reveals that vinasse amendment can impact on ARGs coevolutionary processes [21], suggesting that vinasse amendment is capable to modify the selective pressure upon soil resistome. Nutrient amendment to soils favors copiotrophic microbes [22] and the use of antibiotics to antagonize the growth of competitors and to dominate niches may be employed as a strategy [4]. Therefore, our dataset suggests that vinasse amendment to soils contributes to interference competition within soil microbial communities. Altogether, the results presented here indicate that the spread of resistance against antimicrobial compounds within soil microbial communities by vinasse amendment is a question of concern.

In order to prevent the negative impacts related to bacterial contamination, ethanol mills utilize different compounds with antimicrobial activity such as sulphuric acid, hop crops, chemical biocides and antibiotics [23]. Therefore, it is important to determine if such compounds remain active in vinasse and do not influence resistance genes in soils. Nevertheless, determining antibiotic concentrations in soils can be difficult [24] especially considering the low concentrations used in the industry (in the ppm range). Brazil is one of the major fuel ethanol producers worldwide (more than 23 billions L.year^-1^), yielding 10 to 15 L of vinasse for each L of ethanol produced. This, in turn, results in more than 300 GL of vinasse being applied in 9.7 Mha [1, 2]. It is a considerable amount of vinasse potentially being flushed into soil microbiome in sugarcane croplands. Overall, with our results we were able to provide considerable information to support the hypothesis that vinasse fertirrigation can impact the soil resistome dynamics. We outline the need for experiments to evaluate the dimension of this impact concerning the emergence of resistance in the environment and its potential implications to human health.

## Additional files

Additional file 1: Table S1. Network output Fig. 2a. (CSV 109 kb)

Additional file 2: Table S2. Network output Fig. 2b. (CSV 124 kb)

Additional file 3: Table S3. Network output Fig. 2c. (CSV 117 kb)

Additional file 4: Table S4. Network output Fig. 2d. (CSV 127 kb)

Additional file 5: Table S5. Network output Fig. 2e. (CSV 120 kb)

Additional file 6: Table S6. Network output Fig. 2f. (CSV 98 kb)

Additional file 7: Table S7. Rank table of betweenness centrality. (PDF 128 kb)
