# Supplementary material for: Vinasse fertirrigation alters soil resistome dynamics: an analysis based on metagenomic profiles
Source: BioData Min. 2017 May 23;10:17. doi: 10.1186/s13040-017-0138-4 (PMC5442679; doi:10.1186/s13040-017-0138-4)
Supplement: Supplementary file 7 — Rank table of betweenness centrality. (PDF 128 kb) [file 13040_2017_138_MOESM7_ESM.pdf]

Table S1: Rank of nodes betweenness centrality (BC) predicted by the network models with (+) and without (-) vinasse (V)

7 dap*				157 dap				217 dap			
V-		V+		V-		V+		V-		V+	
**Name**	**BC**	**Name**	**BC**	**Name**	**BC**	**Name**	**BC**	**Name**	**BC**	**Name**	**BC**
RF0092	1.00	RF0095	1.00	RF0142	1.00	RF0103	1.00	RF0070	1.00	RF0082	1.00
RF0155	1.00	RF0104	0.65	RF0168	0.83	RF0078	0.67	RF0020	1.00	RF0100	1.00
RF0134	0.69	RF0057	0.63	RF0119	0.62	RF0064	0.67	RF0015	0.61	RF0085	0.69
RF0017	0.67	RF0126	0.60	RF0129	0.62	RF0128	0.67	RF0025	0.60	RF0134	0.60
RF0152	0.67	RF0083	0.60	RF0163	0.59	RF0169	0.57	RF0115	0.59	RF0173	0.60
RF0004	0.67	RF0148	0.56	RF0165	0.56	RF0065	0.57	RF0052	0.55	RF0061	0.60
RF0060	0.62	RF0159	0.53	RF0141	0.56	RF0077	0.55	RF0121	0.53	RF0123	0.58
RF0148	0.60	RF0129	0.53	RF0094	0.54	RF0095	0.55	RF0030	0.53	RF0131	0.57
RF0010	0.60	RF0007	0.53	RF0032	0.53	RF0036	0.54	RF0068	0.44	RF0026	0.55
RF0136	0.58	RF0127	0.51	RF0166	0.51	RF0125	0.53	RF0125	0.41	RF0142	0.50
RF0088	0.56	RF0136	0.51	RF0152	0.50	RF0050	0.52	RF0169	0.40	RF0128	0.50
RF0173	0.56	RF0019	0.51	RF0065	0.50	RF0163	0.51	RF0128	0.37	RF0168	0.50
RF0086	0.55	RF0162	0.50	RF0079	0.50	RF0069	0.50	RF0043	0.36	RF0153	0.50
RF0062	0.50	RF0059	0.50	RF0002	0.48	RF0129	0.50	RF0149	0.34	RF0116	0.47
RF0075	0.50	RF0146	0.49	RF0033	0.47	RF0160	0.49	RF0044	0.33	RF0075	0.47
RF0055	0.47	RF0135	0.47	RF0173	0.44	RF0136	0.48	RF0009	0.33	RF0120	0.44
RF0080	0.46	RF0055	0.43	RF0097	0.40	RF0031	0.44	RF0144	0.33	RF0006	0.42
RF0158	0.45	RF0113	0.41	RF0085	0.36	RF0155	0.44	RF0173	0.31	RF0051	0.40
RF0018	0.44	RF0163	0.40	RF0047	0.34	RF0098	0.43	RF0100	0.31	RF0084	0.40
RF0040	0.41	RF0075	0.40	RF0003	0.33	RF0006	0.41	RF0116	0.26	RF0015	0.39
RF0117	0.38	RF0156	0.36	RF0172	0.31	RF0048	0.40	RF0053	0.25	RF0133	0.39
RF0094	0.37	RF0072	0.36	RF0139	0.30	RF0063	0.40	RF0135	0.25	RF0147	0.37
RF0172	0.36	RF0090	0.36	RF0038	0.28	RF0162	0.40	RF0090	0.24	RF0125	0.30
RF0126	0.36	RF0100	0.31	RF0156	0.26	RF0156	0.38	RF0033	0.23	RF0164	0.29
RF0048	0.36	RF0147	0.30	RF0162	0.26	RF0085	0.37	RF0153	0.23	RF0011	0.29
RF0119	0.33	RF0014	0.27	RF0114	0.23	RF0140	0.36	RF0012	0.23	RF0151	0.26
RF0021	0.33	RF0042	0.26	RF0087	0.22	RF0040	0.34	RF0040	0.22	RF0137	0.23
RF0131	0.31	RF0116	0.25	RF0169	0.22	RF0093	0.33	RF0019	0.22	RF0002	0.22
RF0078	0.27	RF0124	0.24	RF0158	0.22	RF0003	0.31	RF0027	0.20	RF0056	0.22
RF0149	0.25	RF0167	0.22	RF0101	0.22	RF0060	0.30	RF0103	0.20	RF0152	0.22
RF0108	0.25	RF0145	0.21	RF0007	0.20	RF0011	0.29	RF0007	0.20	RF0054	0.22
RF0013	0.23	RF0103	0.20	RF0084	0.18	RF0112	0.28	RF0147	0.19	RF0029	0.20
RF0051	0.23	RF0157	0.19	RF0005	0.18	RF0034	0.27	RF0035	0.18	RF0030	0.18
RF0045	0.22	RF0144	0.18	RF0082	0.17	RF0057	0.26	RF0136	0.18	RF0109	0.18
RF0137	0.21	RF0048	0.17	RF0041	0.17	RF0052	0.25	RF0160	0.18	RF0161	0.18
RF0025	0.20	RF0039	0.17	RF0130	0.16	RF0119	0.25	RF0014	0.17	RF0104	0.17
RF0053	0.20	RF0107	0.17	RF0125	0.16	RF0087	0.22	RF0168	0.17	RF0092	0.17
RF0133	0.20	RF0155	0.17	RF0004	0.15	RF0076	0.22	RF0127	0.17	RF0079	0.17
RF0028	0.20	RF0022	0.16	RF0167	0.15	RF0165	0.22	RF0018	0.17	RF0060	0.17
RF0068	0.19	RF0114	0.15	RF0080	0.13	RF0088	0.20	RF0167	0.16	RF0072	0.17
RF0145	0.19	RF0053	0.15	RF0022	0.13	RF0017	0.19	RF0017	0.15	RF0027	0.17
RF0074	0.18	RF0158	0.15	RF0133	0.12	RF0082	0.18	RF0172	0.15	RF0127	0.17

RF0161	0.17	RF0128	0.14	RF0053	0.12	RF0107	0.18	RF0042	0.15	RF0063	0.16
RF0120	0.17	RF0121	0.13	RF0061	0.12	RF0150	0.17	RF0139	0.14	RF0159	0.14
RF0081	0.17	RF0021	0.13	RF0063	0.12	RF0132	0.17	RF0028	0.14	RF0034	0.14
RF0057	0.17	RF0015	0.12	RF0144	0.12	RF0124	0.17	RF0059	0.14	RF0004	0.13
RF0033	0.16	RF0101	0.12	RF0111	0.11	RF0068	0.16	RF0162	0.13	RF0129	0.13
RF0043	0.15	RF0133	0.10	RF0023	0.11	RF0026	0.16	RF0048	0.13	RF0090	0.13
RF0083	0.12	RF0169	0.10	RF0050	0.11	RF0005	0.16	RF0112	0.13	RF0097	0.13
RF0036	0.12	RF0034	0.10	RF0068	0.10	RF0045	0.16	RF0087	0.13	RF0146	0.11
RF0160	0.12	RF0003	0.09	RF0036	0.10	RF0067	0.15	RF0164	0.13	RF0150	0.10
RF0167	0.12	RF0085	0.09	RF0120	0.09	RF0074	0.15	RF0058	0.12	RF0171	0.10
RF0087	0.10	RF0017	0.09	RF0088	0.09	RF0094	0.15	RF0082	0.12	RF0016	0.09
RF0132	0.10	RF0038	0.09	RF0020	0.09	RF0066	0.15	RF0131	0.12	RF0023	0.08
RF0029	0.10	RF0011	0.09	RF0024	0.08	RF0167	0.14	RF0064	0.12	RF0139	0.08
RF0006	0.10	RF0153	0.08	RF0098	0.08	RF0084	0.13	RF0054	0.11	RF0145	0.08
RF0116	0.10	RF0074	0.08	RF0052	0.08	RF0168	0.13	RF0129	0.11	RF0017	0.07
RF0099	0.09	RF0109	0.08	RF0078	0.07	RF0042	0.13	RF0003	0.11	RF0022	0.06
RF0105	0.09	RF0139	0.08	RF0083	0.07	RF0030	0.12	RF0081	0.10	RF0119	0.06
RF0059	0.08	RF0016	0.07	RF0015	0.07	RF0173	0.12	RF0083	0.09	RF0028	0.06
RF0122	0.08	RF0020	0.07	RF0116	0.07	RF0139	0.12	RF0051	0.09	RF0076	0.06
RF0096	0.08	RF0160	0.07	RF0135	0.07	RF0115	0.11	RF0130	0.09	RF0106	0.06
RF0127	0.06	RF0151	0.07	RF0157	0.06	RF0037	0.11	RF0106	0.09	RF0113	0.06
RF0019	0.06	RF0054	0.05	RF0113	0.05	RF0058	0.11	RF0134	0.08	RF0130	0.06
RF0163	0.06	RF0066	0.05	RF0030	0.05	RF0022	0.11	RF0141	0.08	RF0101	0.05
RF0106	0.06	RF0028	0.05	RF0149	0.04	RF0105	0.10	RF0174	0.08	RF0050	0.05
RF0020	0.04	RF0030	0.05	RF0027	0.04	RF0055	0.09	RF0124	0.07	RF0162	0.03
RF0090	0.04	RF0134	0.04	RF0127	0.03	RF0133	0.09	RF0011	0.07	RF0099	0.02
RF0026	0.04	RF0137	0.04	RF0001	0.03	RF0116	0.08	RF0061	0.07	RF0058	0.01
RF0128	0.04	RF0027	0.04	RF0013	0.03	RF0028	0.07	RF0111	0.06	RF0088	0.01
RF0174	0.04	RF0052	0.04	RF0037	0.03	RF0016	0.07	RF0047	0.06	RF0169	0.01
RF0003	0.04	RF0063	0.04	RF0148	0.02	RF0157	0.06	RF0034	0.06	RF0059	0.01
RF0118	0.03	RF0005	0.04	RF0126	0.02	RF0024	0.05	RF0098	0.06	RF0163	0.01
RF0050	0.02	RF0049	0.03	RF0026	0.02	RF0008	0.05	RF0145	0.06	RF0020	0.01
RF0063	0.01	RF0036	0.03	RF0100	0.01	RF0101	0.05	RF0094	0.06	RF0105	0.00
RF0015	0.01	RF0084	0.03	RF0112	0.01	RF0130	0.05	RF0046	0.06	RF0009	0.00
RF0034	0.01	RF0056	0.03	RF0146	0.01	RF0164	0.04	RF0152	0.06	RF0021	0.00
RF0162	0.01	RF0105	0.02	RF0059	0.01	RF0032	0.04	RF0037	0.04	RF0080	0.00
RF0072	0.01	RF0131	0.02	RF0128	0.01	RF0108	0.03	RF0057	0.04	RF0081	0.00
RF0091	0.01	RF0165	0.02	RF0099	0.01	RF0171	0.03	RF0101	0.03	RF0049	0.00
RF0084	0.01	RF0099	0.02	RF0122	0.01	RF0134	0.03	RF0091	0.03	RF0036	0.00
RF0097	0.01	RF0068	0.02	RF0039	0.00	RF0013	0.02	RF0161	0.03	RF0048	0.00
RF0114	0.00	RF0044	0.01	RF0151	0.00	RF0100	0.02	RF0119	0.03	RF0068	0.00
RF0166	0.00	RF0033	0.01	RF0134	0.00	RF0075	0.02	RF0099	0.03	RF0042	0.00
RF0030	0.00	RF0026	0.01	RF0014	0.00	RF0149	0.02	RF0029	0.03	RF0067	0.00
RF0156	0.00	RF0130	0.01	RF0137	0.00	RF0148	0.02	RF0026	0.02	RF0031	0.00
RF0027	0.00	RF0108	0.01	RF0034	0.00	RF0012	0.02	RF0063	0.02	RF0096	0.00
RF0085	0.00	RF0152	0.01	RF0117	0.00	RF0009	0.02	RF0084	0.01	RF0126	0.00

RF0113	0.00	RF0041	0.01	RF0106	0.00	RF0158	0.01	RF0055	0.01	RF0140	0.00
RF0054	0.00	RF0132	0.01	RF0054	0.00	RF0025	0.01	RF0126	0.01	RF0037	0.00
RF0164	0.00	RF0040	0.00	RF0131	0.00	RF0027	0.01	RF0158	0.01	RF0118	0.00
RF0061	0.00	RF0166	0.00	RF0161	0.00	RF0023	0.01	RF0109	0.01	RF0132	0.00
RF0168	0.00	RF0043	0.00	RF0072	0.00	RF0061	0.01	RF0132	0.01	RF0038	0.00
RF0142	0.00	RF0062	0.00	RF0016	0.00	RF0038	0.01	RF0041	0.01	RF0091	0.00
RF0151	0.00	RF0058	0.00	RF0051	0.00	RF0099	0.01	RF0157	0.00	RF0040	0.00
RF0111	0.00	RF0122	0.00	RF0164	0.00	RF0152	0.01	RF0069	0.00	RF0086	0.00
RF0146	0.00	RF0061	0.00	RF0006	0.00	RF0137	0.01	RF0096	0.00	RF0005	0.00
RF0008	0.00	RF0051	0.00	RF0010	0.00	RF0126	0.01	RF0086	0.00	RF0053	0.00
RF0077	0.00	RF0087	0.00	RF0090	0.00	RF0114	0.01	RF0120	0.00	RF0144	0.00
RF0104	0.00	RF0006	0.00	RF0108	0.00	RF0039	0.01	RF0142	0.00	RF0074	0.00
RF0067	0.00	RF0008	0.00	RF0104	0.00	RF0121	0.00	RF0031	0.00	RF0112	0.00
RF0109	0.00	RF0092	0.00	RF0147	0.00	RF0086	0.00	RF0039	0.00	RF0156	0.00
RF0129	0.00	RF0050	0.00	RF0096	0.00	RF0106	0.00	RF0067	0.00	RF0033	0.00
RF0023	0.00	RF0173	0.00	RF0035	0.00	RF0161	0.00	RF0097	0.00	RF0083	0.00
RF0130	0.00	RF0091	0.00	RF0076	0.00	RF0051	0.00	RF0092	0.00	RF0066	0.00
RF0044	0.00	RF0067	0.00	RF0028	0.00	RF0145	0.00	RF0166	0.00	RF0158	0.00
RF0065	0.00	RF0070	0.00	RF0118	0.00	RF0033	0.00	RF0105	0.00	RF0019	0.00
RF0140	0.00	RF0069	0.00	RF0040	0.00	RF0117	0.00	RF0024	0.00	RF0124	0.00
RF0041	0.00	RF0023	0.00	RF0048	0.00	RF0172	0.00	RF0062	0.00	RF0064	0.00
RF0069	0.00	RF0174	0.00	RF0081	0.00	RF0090	0.00	RF0117	0.00	RF0044	0.00
RF0001	0.00	RF0004	0.00	RF0044	0.00	RF0079	0.00	RF0016	0.00	RF0167	0.00
RF0141	0.00	RF0094	0.00	RF0160	0.00	RF0081	0.00	RF0156	0.00	RF0111	0.00
RF0035	0.00	RF0024	0.00	RF0046	0.00	RF0120	0.00	RF0151	0.00	RF0046	0.00
RF0115	0.00	RF0076	0.00	RF0064	0.00	RF0104	0.00	RF0078	0.00	RF0157	0.00
RF0165	0.00	RF0143	0.00	RF0062	0.00	RF0092	0.00	RF0146	0.00	RF0103	0.00
RF0100	0.00	RF0077	0.00	RF0124	0.00	RF0047	0.00	RF0066	0.00	RF0148	0.00
RF0037	0.00	RF0098	0.00	RF0153	0.00	RF0041	0.00	RF0163	0.00	RF0165	0.00
RF0011	0.00	RF0123	0.00	RF0171	0.00	RF0146	0.00	RF0006	0.00	RF0014	0.00
RF0009	0.00	RF0013	0.00	RF0115	0.00	RF0144	0.00	RF0150	0.00	RF0117	0.00
RF0159	0.00	RF0118	0.00	RF0011	0.00	RF0159	0.00	RF0118	0.00	RF0172	0.00
RF0002	0.00	RF0081	0.00	RF0075	0.00	RF0053	0.00	RF0140	0.00	RF0174	0.00
RF0093	0.00	RF0089	0.00	RF0103	0.00	RF0141	0.00	RF0079	0.00	RF0057	0.00
RF0157	0.00	RF0119	0.00	RF0140	0.00	RF0004	0.00	RF0074	0.00	RF0062	0.00
RF0046	0.00	RF0045	0.00	RF0021	0.00	RF0014	0.00	RF0036	0.00	RF0114	0.00
RF0103	0.00	RF0047	0.00	RF0091	0.00	RF0113	0.00	RF0056	0.00	RF0135	0.00
RF0032	0.00	RF0037	0.00	RF0174	0.00	RF0083	0.00	RF0171	0.00	RF0077	0.00
RF0171	0.00	RF0120	0.00	RF0066	0.00	RF0046	0.00	RF0159	0.00	RF0055	0.00
RF0123	0.00	RF0031	0.00	RF0060	0.00	RF0056	0.00	RF0010	0.00	RF0039	0.00
RF0124	0.00	RF0060	0.00	RF0121	0.00	RF0123	0.00	RF0013	0.00	RF0007	0.00
RF0154	0.00	RF0142	0.00	RF0042	0.00	RF0015	0.00	RF0113	0.00	RF0098	0.00
RF0076	0.00	RF0093	0.00	RF0025	0.00	RF0109	0.00	RF0165	0.00	RF0107	0.00
RF0153	0.00	RF0088	0.00	RF0056	0.00	RF0072	0.00	RF0080	0.00	RF0154	0.00
RF0112	0.00	RF0010	0.00	RF0105	0.00	RF0111	0.00	RF0075	0.00	RF0122	0.00
RF0121	0.00	RF0112	0.00	RF0109	0.00	RF0142	0.00	RF0077	0.00	RF0136	0.00

RF0031	0.00	RF0082	0.00	RF0136	0.00	RF0118	0.00	RF0032	0.00	RF0010	0.00
RF0101	0.00	RF0035	0.00	RF0093	0.00	RF0102	0.00	RF0088	0.00	RF0160	0.00
RF0150	0.00	RF0012	0.00	RF0107	0.00	RF0174	0.00	RF0023	0.00	RF0008	0.00
RF0079	0.00	RF0172	0.00	RF0086	0.00	RF0080	0.00	RF0005	0.00	RF0078	0.00
RF0144	0.00	RF0018	0.00	RF0055	0.00	RF0062	0.00	RF0022	0.00	RF0065	0.00
RF0007	0.00	RF0149	0.00	RF0031	0.00	RF0035	0.00	RF0114	0.00	RF0166	0.00
RF0056	0.00	RF0025	0.00	RF0009	0.00	RF0018	0.00	RF0002	0.00	RF0094	0.00
RF0125	0.00	RF0009	0.00	RF0067	0.00	RF0127	0.00	RF0104	0.00	RF0052	0.00
RF0064	0.00	RF0125	0.00	RF0123	0.00	RF0147	0.00	RF0060	0.00	RF0013	0.00
RF0058	0.00	RF0078	0.00	RF0012	0.00	RF0059	0.00	RF0108	0.00	RF0024	0.00
RF0147	0.00	RF0150	0.00	RF0074	0.00	RF0131	0.00	RF0008	0.00	RF0141	0.00
RF0042	0.00	RF0140	0.00	RF0058	0.00	RF0049	0.00	RF0137	0.00	RF0043	0.00
RF0022	0.00	RF0079	0.00	RF0029	0.00	RF0122	0.00			RF0087	0.00
RF0071	0.00	RF0080	0.00	RF0102	0.00	RF0007	0.00			RF0070	0.00
RF0047	0.00	RF0111	0.00	RF0143	0.00	RF0054	0.00			RF0001	0.00
RF0012	0.00	RF0065	0.00	RF0092	0.00	RF0021	0.00			RF0003	0.00
RF0005	0.00	RF0029	0.00	RF0159	0.00	RF0151	0.00				
RF0139	0.00	RF0117	0.00			RF0091	0.00				
RF0039	0.00	RF0106	0.00			RF0044	0.00				
RF0016	0.00	RF0161	0.00			RF0166	0.00				
RF0095	0.00	RF0141	0.00			RF0089	0.00				
RF0098	0.00	RF0032	0.00			RF0096	0.00				
RF0052	0.00					RF0019	0.00				

*dap: days after planting
